# The slopes of sub-barrier heavy-ion fusion excitation functions shed light on the dynamics of quantum tunnelling

**DOI:** 10.1038/s41598-024-63107-7

**Published:** 2024-06-04

**Authors:** A. M. Stefanini, G. Montagnoli, M. Del Fabbro, L. Corradi, E. Fioretto, S. Szilner

**Affiliations:** 1 INFN, Laboratori Nazionali di Legnaro, I-35020 Legnaro, Italy; 2 Dipartimento di Fisica e Astronomia, Università di Padova and INFN, Padova, Italy; 3 Ruđer, Bošković Institute, Zagreb, Croatia

**Keywords:** Physics, Nuclear physics

## Abstract

Quantum tunnelling plays a crucial role in heavy-ion fusion reactions at sub-barrier energies, especially in the context of nuclear physics and astrophysics. The nuclear structure of the colliding nuclei and nucleon transfer processes represent intrinsic degrees of freedom. They are coupled to the relative ion motion and, in general, increase the probability of tunnelling. The influence of couplings to nucleon transfer channels relatively to inelastic excitations, on heavy-ion fusion cross sections, is one of the still open problems in this field. We present a new analysis of several systems, based on the combined observation of the energy-weighted excitation functions $E\sigma$ in relation to their first energy derivatives $d(E\sigma )/dE$. The relation between $d(E\sigma )/dE$ and $E\sigma$ removes the basic differences due to the varying Coulomb barrier when comparing different systems. We show that, depending on the nuclear structure and/or the presence of strong transfer channels, this representation reveals characteristic features below the barrier. The possible presence of cross section oscillations makes this analysis less clear for light- or medium-light systems.

## Introduction

Quantum tunneling is a quantum mechanical phenomenon where particles can pass through energy barriers that classical physics would predict to be insurmountable. The basic idea is that particles, such as nuclei, can “tunnel” through potential energy barriers even when their energy is less than the height of the barrier. When considering sub-barrier heavy-ion fusion, quantum tunneling in the presence of intrinsic degrees of freedom has to be considered, and we enter a domain where the interplay between tunneling and intrinsic degrees of freedom leads to rich and diverse physical phenomena presented and discussed in recent review articles^1–4^.

Extensive experimental and theoretical research in this field has additionally identified the sub-barrier hindrance effect^5^ at far sub-barrier energies, shortly described in Appendix 1, and its possible consequences in astrophysics. The structure of the colliding nuclei is known to produce large enhancements of the fusion excitation functions, and evidence of strong isotopic effects are observed, that is, fusion excitation functions of nearby systems may differ substantially in magnitude and shape.

Such experimental evidence was successfully reproduced by the coupled-channels (CC) model^6–8^ that is based on the close connection existing between the low-lying collective excitations of the two colliding nuclei, and the near- and sub-barrier fusion cross sections, so that fusion barrier distributions are produced by couplings to such excitations^9^. Similar distributions can be obtained from large-angle quasi-elastic scattering. It was pointed out that extra absorption into a large number of non-collective inelastic channels leads to a smearing of the barrier distribution^10^, see also the more recent work^11^ and the Refs. therein.

However, a basic unsettled problem is the possible influence of couplings to nucleon transfer modes. The importance of transfer couplings is often deduced by observing that couplings to only inelastic excitations are not sufficient to reproduce the measured enhancements of the cross sections, or by comparing the excitation functions of different systems using reduced energy and cross section scales, where the height and the radius of the barrier are used (see also Ref.^12^). These procedures are of course model-dependent, thus implying that uncertainties of various types may be introduced.

In this work, we present a new analysis of the behaviour of a number of systems. We propose the combined observation of the energy-weighted excitation functions $E\sigma$ with respect to their first energy derivatives $d(E\sigma )/dE$. In the following the derivative $\frac{d(E\sigma _{fus})}{dE}$ will be named “slope”. We will see that this representation helps understanding the underlying physics on the plain basis of experimental data (and removes the dependence of data on the barrier height). For all discussed systems, theoretical calculations, most of which use the CC model, are not reported here and can be found in the original articles cited in the References.

## Basic concepts

In Appendix 2 the Wong’s formula^13,14^ for charged-particle fusion in nuclear reactions is briefly recalled. The energy derivative of that expression at sub-barrier energies is given by

1 $$\begin{aligned} \frac{d(E\sigma _{fus})}{dE}=\frac{\hbar \omega R_b^2}{2}exp\left[ \frac{2\pi }{\hbar \omega } (E-V_b)\right] \frac{2\pi }{\hbar \omega }=\frac{2\pi }{\hbar \omega }E\sigma _{fus} \end{aligned}$$

where $R_b$ is the barrier radius and $\omega$ is the frequency related to the parabolic barrier. Therefore, in the Wong approximation, the sub-barrier excitation function and its slope are proportional to each other, related by the quantity $2\pi /\hbar \omega$, which depends on the barrier width, but not on its height. In a plot of $d(E\sigma _{fus})/dE$ vs $E\sigma _{fus}$ the angular coefficient is $2\pi /\hbar \omega$, and a steeper slope will be associated to a thicker barrier.

$2\pi /\hbar \omega$ equals the logarithmic derivative of $E\sigma _{fus}$ since

2 $$\begin{aligned} \frac{2\pi }{\hbar \omega }=\frac{1}{E\sigma _{fus}} \frac{d(E\sigma _{fus})}{dE}=\frac{dln(E\sigma _{fus})}{dE} \end{aligned}$$

When one introduces the CC model of Dasso et al.^6–8,15^, a splitting of the original single barrier takes place as a consequence of couplings of the entrance channel to inelastic or transfer channels, and a fusion barrier distribution is produced.

**Figure 1:**
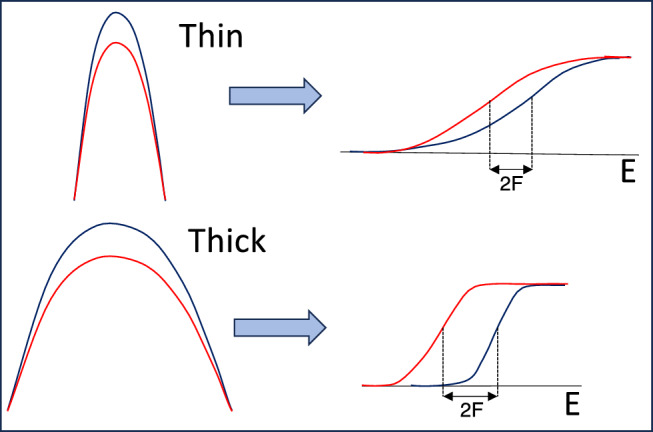
Pictorial view of thin (thick) barriers and (on the right) of the corresponding transmission functions as expected from the CC model.

In the simplified case of one coupled channel, let *F* be its coupling strength near the barrier top (a typical value of *F* for heavy-ion fusion is $\simeq$1 MeV). The separation between the two barriers produced by the coupling is then 2*F* (see Fig. 1). Whether we customarily call the barrier “thin” or a “thick”, depends on comparing *F* to the parameter $\epsilon =\hbar \omega$/$2\pi$ characterising the barrier width (see Appendix 2). For cases where $\epsilon \sim 2F$ we have a thin barrier, while when $\epsilon <2F$, the barrier is thick^15^.

While the total transmission function is classically given by a sum of step functions, in the case of a thin barrier the smoothing due to quantum effects tends to wash out the splitting and produce a smooth transmission function, that is, a small derivative of the excitation function (see again Fig. 1). The opposite is true with a thicker barrier when the splitting of barrier heights is essentially preserved even after accounting for quantum effects. The tunnelling probability at low energies is small, leading to a steep excitation function.

**Figure 2:**
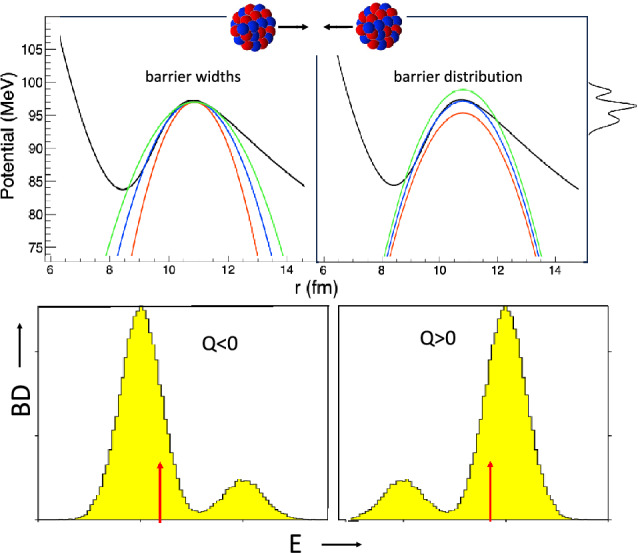
(top panel) Qualitative picture of two situations in a heavy-ion collision. (left) Coulomb barriers of different widths, (right) example of a barrier distribution with 3 peaks, with its projection on the potential axis. The black line is the ion-ion potential following Akyüz-Winther parametrization in a wider range of radii, calculated for ^58^Ni + ^58^Ni^16^. (bottom panel) Simplified view of the barrier distributions predicted by the CC model^6–8,15^ for coupling to one $Q<0$ channel (left) and one $Q>0$ channel (right). The arrows qualitatively mark the location of the uncoupled barrier in the two cases.

Figure 2 (top panel) is a qualitative representation of parabolic barriers with different widths (left panel), and of the barrier distribution produced by couplings (right).

We consider now coupled channels with positive and negative Q-values ($Q>0$ and $Q<0$), and we indicate again their coupling strength with *F*. In either case enhancements of the transmission function will be produced vs energy^6^, however, different features will be observed. The bottom panel of Fig. 2 qualitatively shows the two fusion barrier distributions predicted by the CC model, by assuming that *F* is significantly smaller than |*Q*|, as it occurs in most cases.

The barrier is reduced appreciably by the coupling interaction when $Q>0$, even if only a small fraction of the incident flux reaches this lower barrier. The barrier is less lowered by couplings to channels with $Q<0$, but most of the flux faces this slightly lower barrier and the net effect is to produce a simple shift in barrier height. In other words, the transmission function will be smoother for $Q>0$ couplings, with respect to $Q<0$ couplings, in particular when logarithmic plots vs energy are observed. The lowest effective barrier will have the largest (smallest) weight for negative (positive) *Q* values, and $d(E\sigma )/dE$ will be correspondingly larger (smaller).

An analogy exists between the effects of couplings to $Q>0$ reaction channels and the case of a thin barrier in the one-dimensional potential barrier limit. Indeed, both these (obviously physically different) situations lead to a smoother fusion excitation function at low energies (a smaller $d(E\sigma )/dE$). We shall exploit this formal similarity in the analysis of several heavy-ion systems presented in the following Sections, that is purely based on experimental data.

## Relevant experimental evidences

**Figure 3:**
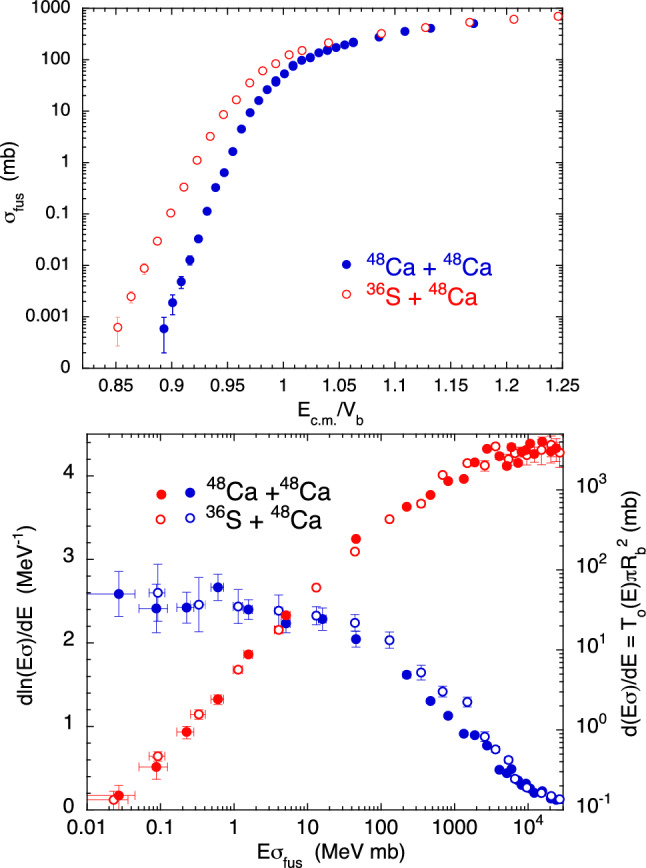
(top panel) Experimental fusion excitation functions. (bottom panel) Plot of $d[ln(E\sigma )]/dE$ (blue dots) and of $d(E\sigma )/dE$ (red dots) vs $E\sigma$ for ^48^Ca, ^36^S + ^48^Ca^17,18^. The right ordinate of this bottom panel is proportional to the s-wave transmission coefficient and the square of the barrier radius. In this figure and the following ones, the reported experimental errors are statistical uncertainties, and most of them are smaller than the data symbols in several cases.

In this Section, we present and discuss the behaviour of several representative heavy-ion systems where nuclear structure and/or nucleon transfer reactions play significant roles in the dynamics of sub-barrier fusion. In Fig. 3 and all following ones, the derivatives and the logarithmic derivatives of the excitation functions have been obtained from the measured sets of data, using the incremental ratio between successive or over-successive energy points, as customarily done in analyses of this kind^1,4^.

### Magic and closed-shell nuclei

The two systems ^48^Ca,^36^S + ^48^Ca were investigated in Refs.^17,18^. The top panel of Fig. 3 shows their excitation functions. The energy scale is normalized to the Coulomb barrier, as obtained from the Akyüz-Winther potential^16^. The CC calculations reproducing those data were performed using the code CCFULL^19^, and are reported in the original papers.

For the same two cases, we report in the bottom panel the slope $d(E\sigma )/dE$ and the logarithmic derivative $dln[(E\sigma )]/dE$ of the excitation functions as a function of $E\sigma$. In this representation, trivial Coulomb barrier height differences between the two systems are eliminated to a large extent.

With a parabolic barrier and using the approximations reported in Appendix 2, the slope $d(E\sigma )/dE$ turns out to be proportional to the s-wave penetrability as shown in detail in Ref.^2^, that is

3 $$\begin{aligned} \frac{d(E\sigma _{fus})}{dE}\simeq \pi R^2_b T_0(E) \end{aligned}$$

This is reported in the right ordinate of Fig. 3 (bottom panel). The colliding nuclei are very stiff, and we see that the data sets for the two systems are very close to each other. This suggests that the corresponding barriers have approximately the same width. We point out that in both cases, the measured barrier distributions are dominated by a single strong peak^17,18^. The slopes saturate at high energies where the transmission coefficient $T_o$ is one ($R_b$ only weakly depends on *E* in the measured energy ranges).

The behaviour of the two low-energy logarithmic derivatives clearly confirms the strong similarity between the two systems. The bottom panel of Fig. 3 indicates that, after a sharp increase just below the Coulomb barrier, the derivatives level off and become pretty constant with decreasing energy. ^48^Ca,^36^S + ^48^Ca give us a good starting point to look at the behaviour of other cases where inelastic excitations and/or nucleon transfer channels are expected (or already known) to have a strong influence on the sub-barrier fusion cross sections.

### Couplings to $Q>$0 nucleon transfer channels

**Figure 4:**
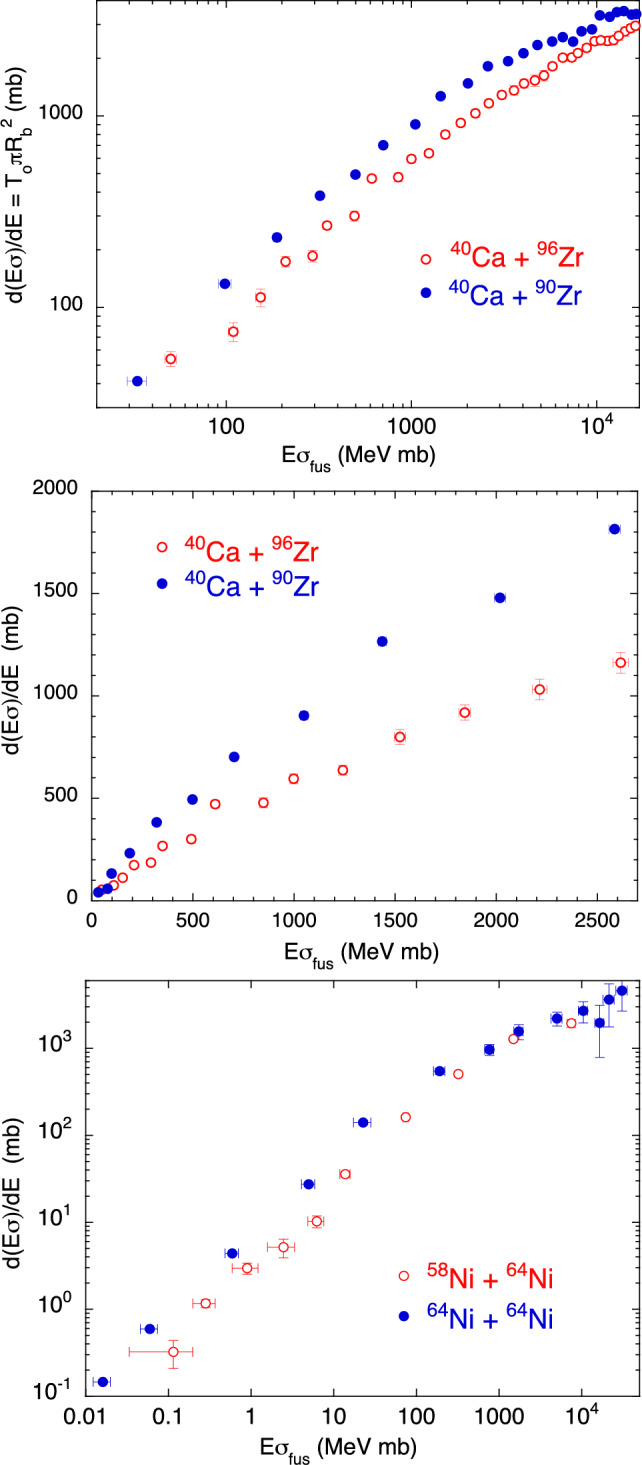
Two-dimensional plot $d(E\sigma )/dE$ vs $E\sigma$ for ^40^Ca + ^90,96^Zr^20,22,24^ (top and center panels), and ^58,64^Ni + ^64^Ni^21,23^ (bottom panel).

We now consider the two pairs of systems reported in the panels of Fig. 4. It is well established that in the two cases ^40^Ca + ^96^Zr^20^ and ^58^Ni + ^64^Ni^21^ nucleon transfer couplings with $Q>0$ produce large cross section enhancements. This is reflected in the different behaviour with respect to ^40^Ca + ^90^Zr^22^ and ^64^Ni + ^64^Ni^23^, respectively. It is evident from Fig. 4 that for both pairs, the system where transfer couplings are dominant, displays a smaller derivative $d(E\sigma )/dE$ with respect to the other case (the barrier in the one-dimensional limit is thinner). This simulates a wider barrier distribution (extending to lower energies, see Fig. 2, lower panel, right) as actually produced by channel couplings, which leads to the observed large cross section enhancement in the sub-barrier region. The linear plot of the center panel makes even more clear the difference between the two systems ^40^Ca + ^96,90^Zr. As introduced in the previous Section, the sub-barrier slope and the excitation function are expected to be proportional to each other. This is what we observe in the figure in a wide $E\sigma$ range, separately for each system, and the dissimilarity with respect to the two cases of Fig. 3 is obvious.

**Figure 5:**
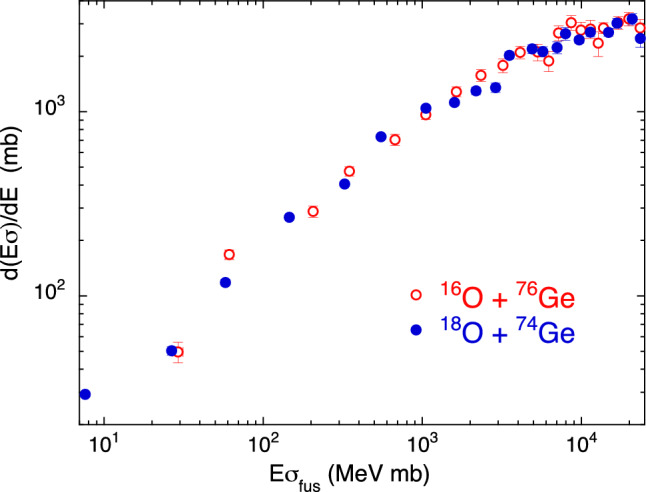
Two-dimensional plot $d(E\sigma )/dE$ vs $E\sigma$ for ^16^O + ^76^Ge and ^18^O + ^74^Ge^25^.

We next discuss the couple of systems ^16^O + ^76^Ge and ^18^O + ^74^Ge^25^. The pick-up of two neutrons changes the first one to the second, and viceversa. The corresponding ground state *Q*-values are -3.75 MeV and +3.75 MeV, respectively. In the original article, it was concluded that no fusion enhancement due to the positive *Q*-value of two-neutron transfer for ^18^O + ^74^Ge is observed as compared with ^16^O + ^76^Ge, on the basis of CC calculations and of the observation of the two reduced excitation functions. Fig. 5 (based simply on experimental data) confirms that conclusion because no significant difference can be observed between the two systems. This may be due to weak transfer coupling strengths in both cases, since ^16,18^O are light nuclei.

**Figure 6:**
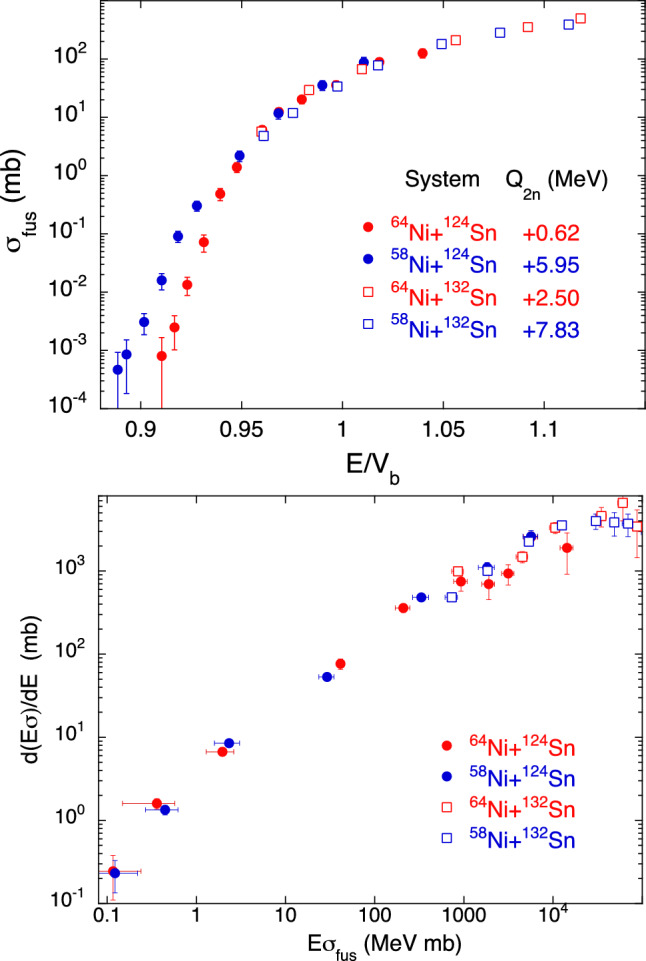
(top panel) Fusion excitation functions for ^58,64^Ni + ^124^Sn^28^ and for ^132^Sn + ^58,64^Ni^26,27^. The abscissa is the energy with respect to the Akyüz-Winther Coulomb barrier. This panel is adapted from Ref.^4^. (bottom panel) Representation of $d(E\sigma )/dE$ vs $E\sigma$ for the same systems.

As a further relevant case, we show in Fig. 6 the behaviour of several Ni + Sn systems. Two of them, ^58,64^Ni + ^132^Ni^26,27^, were studied at Oak Ridge some years ago, using the radioactive ^132^Ni beam. The fusion cross sections were only measured down to some mb (upper panel), where the effect of possible neutron transfer couplings with $Q>$0 is anyway still negligible, as remarked in Ref.^28^. This is confirmed in the representation $d(E\sigma )/dE$ vs $E\sigma$ of the lower panel.

The two excitation functions of ^58,64^Ni + ^124^Ni^28^ (upper panel) reported in a reduced energy scale would qualitatively suggest that transfer couplings produce a much larger cross section enhancement for ^58^Ni + ^124^Ni at sub-barrier energies.

However, the trends reported in the lower panel do not support that evidence, because the data points for the two systems overlap to a large extent in the full energy range below the barrier. This is a model-free support to the conclusions of Ref.^28^ where, based on detailed CC calculations, the strong contribution of inelastic couplings was pointed out, leaving little space to the fusion enhancement produced by transfer. This is typically observed in heavy systems where inelastic modes are dominant. In Ref.^28^, it was pointed out that the contribution from transfer is weaker for ^64^Ni + ^124^Ni due to the smaller transfer *Q*-values (see the representative $Q_{2n}$ in the upper panel of Fig. 6).

**Figure 7:**
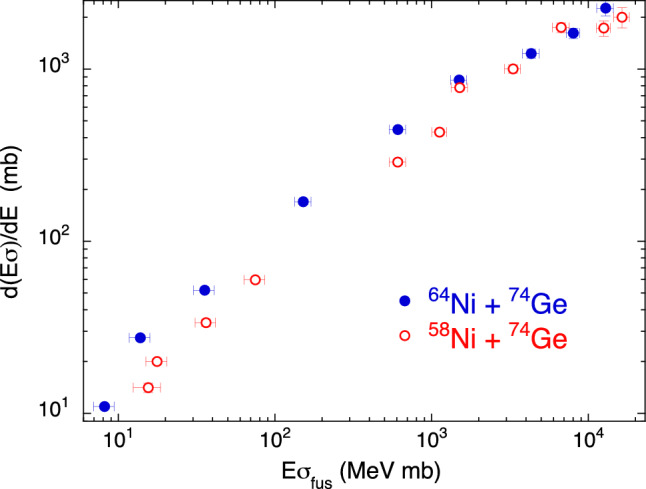
Two-dimensional plot $d(E\sigma )/dE$ vs $E\sigma$ for ^64,58^Ni + ^74^Ge^29^.

Finally, Fig. 7 shows the situation for the medium-heavy systems ^64,58^Ni + ^74^Ge^29^. Here the vibrational structure of ^74^Ge is important, however the concurrent influence of strong $Q>0$ neutron pick-up couplings in ^58^Ni + ^74^Ge only, produces a clearly different trend for this system. Its excitation function has a less steep slope, even if ^58^Ni is more rigid than ^64^Ni.

### Couplings to inelastic excitations

**Figure 8:**
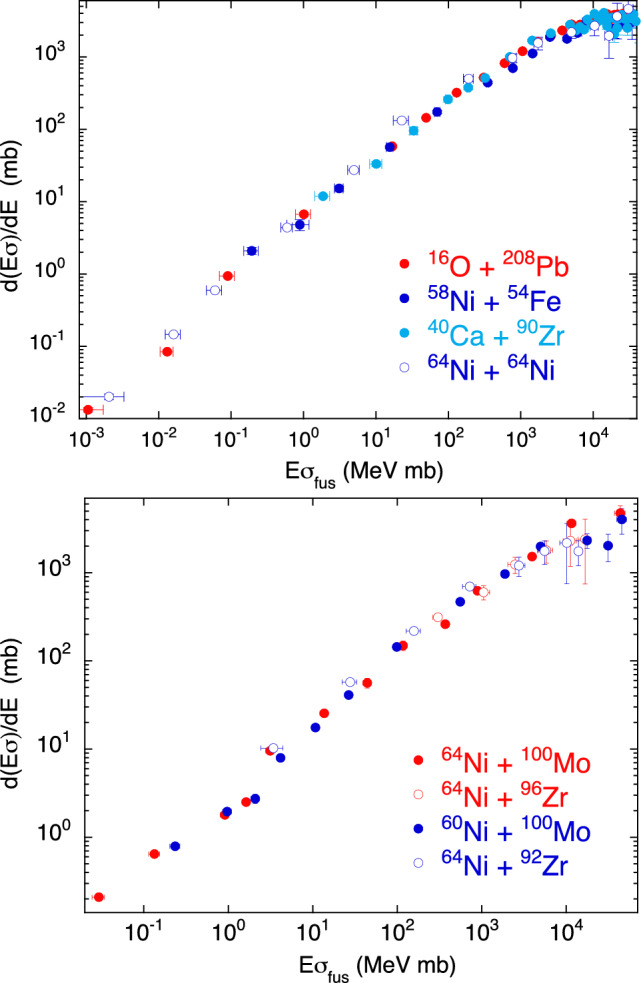
(top panel) Two-dimensional plot $d(E\sigma )/dE$ vs $E\sigma$ for ^16^O + ^208^Pb^30,31^, ^58^Ni + ^54^Fe^32^, ^40^Ca + ^90^Zr^24,33^ and ^64^Ni + ^64^Ni^23^. (bottom panel) The same representation for ^64,60^Ni + ^100^Mo^34,35^ and ^64^Ni + ^92,96^Zr^36^.

In the case of couplings to inelastic excitations the *Q*-values are obviously negative. In the top panel of Fig. 8 we show the behaviour of the four systems ^16^O + ^208^Pb^30,31^, ^58^Ni + ^54^Fe^32^, ^40^Ca + ^90^Zr^24,33^ and of ^64^Ni + ^64^Ni^23^. They are very different cases both from the point of view of mass asymmetry and from that of nuclear structure. It is however common to them a rather stiff structure, and in the representation of $d(E\sigma )/dE$ vs $E\sigma$ the data sets for the four cases are remarkably coincident.

We plot in Fig. 8 (bottom panel) the behaviour of four other systems where ^64,60^Ni are involved. We know that the near- and sub-barrier fusion of ^64,60^Ni + ^100^Mo are dominated by couplings to the low-lying quadrupole excitation of ^100^Mo^34,35^, up to the fourth phonon level, while for the two other cases ^64^Ni + ^92,96^Zr^36^ the important coupled channels are the (weak) quadrupole vibration of ^92^Zr and the (strong) octupole vibration of ^96^Zr. In spite of their different nature, all these vibrational modes produce fusion excitation functions that, in the $d(E\sigma )/dE$ vs $E\sigma$ representation, have an evident overlap. As indicated at the beginning of this Section, this is a consequence of the negative *Q*-values of all relevant coupled channels.

### Medium-light systems

Medium-light systems are being investigated, with the purpose of allowing a convincing extrapolation to the lighter systems of astrophysical interest like C+C, C+O and O+O, for which challenging measurements are needed at the very low energies typical of astrophysics.

**Figure 9:**
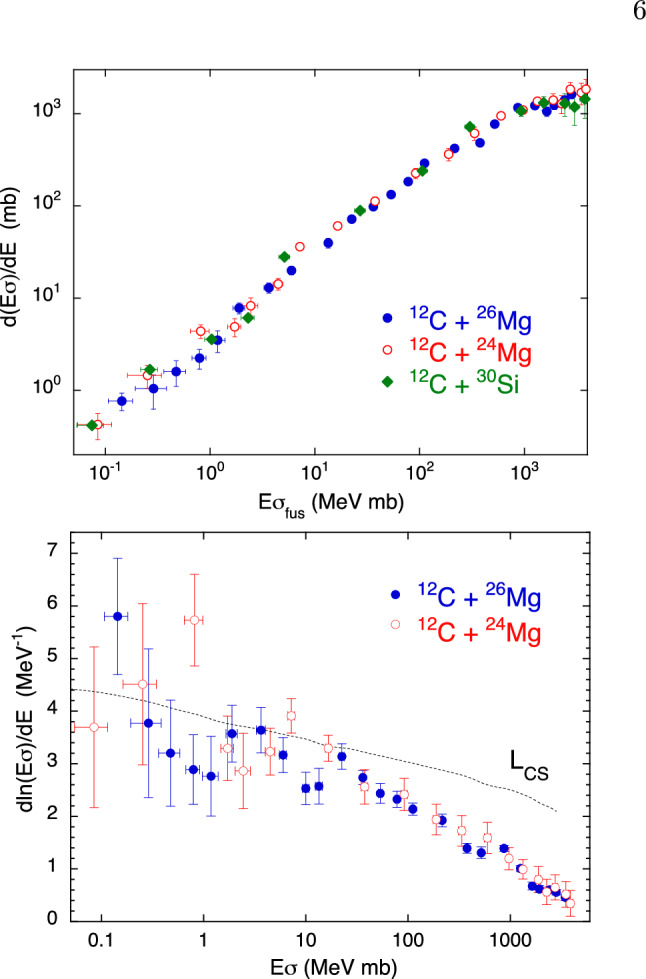
Two-dimensional plots $d(E\sigma )/dE$ vs $E\sigma$ (top panel) and $d[ln(E\sigma )]/dE$ vs $E\sigma$ (bottom panel) for ^26,24^Mg + ^12^C^37,38^. The top panel reports also the trend of ^30^Si + ^12^C^39^. The line indicated with $L_{CS}$ marks the logarithmic derivative values corresponding to a constant astrophysical *S* factor, for different energies (see Ref.^23^).

We analyze here the two cases of ^26,24^Mg + ^12^C, whose fusion excitation functions have been recently measured down to a few $\mu$b^37,38^. Their behaviour in the representation $d(E\sigma )/dE$ vs $E\sigma$ is similar, as shown in the top panel of Fig. 9, though the trends are not smooth for the two systems. The same is true for ^30^Si + ^12^C^39^, as one sees in the same panel. We point out that ^30^Si is a spherical nucleus, while ^26,24^Mg have a permanent prolate deformation, and that all one- and two-nucleon transfer channels have negative *Q*-values for the three systems (small influence on fusion expected).

On the other hand, in the bottom panel, the two logarithmic derivatives $d[ln(E\sigma )]/dE$ (in a linear scale), even accounting for the rather large experimental errors for ^24^Mg + ^12^C, show various oscillations (see Ref.^37^ for a detailed discussion). ^30^Si + ^12^C is not reported here, because of the smaller number of available data points. In the original papers it was remarked that fusion hindrance appears at different cross section levels for the two Mg + C systems as well as for ^30^Si + ^12^C, on the basis of the comparison with CC calculations using a Woods-Saxon potential. However, we note that the large uncertainties for ^24^Mg + ^12^C and the presence of oscillations weaken this statement.

In any case, the representation $d(E\sigma )/dE$ vs $E\sigma$ does not give relevant information on channel coupling effects in the present cases.

Also the lighter systems ^16^O + ^16^O, ^12^C^3^ of astrophysical interest present oscillations of the derivative $d(E\sigma )/dE$ above as well as below the barrier. Above the barrier, they are probably due to the overcoming of successive centrifugal barriers well spaced in energy^40^. Below the barrier, they might originate from the low level density of the compound nuclei^3^, as it is probably the case also for ^26,24^Mg + ^12^C.

### Overall systematics

**Figure 10:**
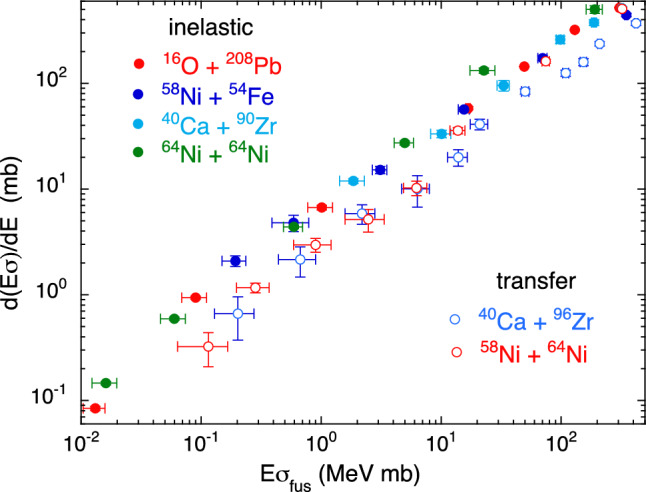
Two-dimensional plot $d(E\sigma )/dE$ vs $E\sigma$ for several systems where either couplings to inelastic modes are dominant (up left), or to transfer couplings are very strong (bottom right).

The behaviour of several analyzed systems is grouped together in Fig. 10. A few of them are recognized cases where couplings to transfer channels are the most important ingredients of the sub-barrier fusion excitation functions (^40^Ca + ^96^Zr and ^58^Ni + ^64^Ni). In the other cases the couplings to low-lying inelastic modes dominate the fusion dynamics.

Two well separated groups of systems are evident, matching the nature of the dominant couplings. This is remarkable, when considering that the various systems were measured with different set-ups and in different laboratories. The slopes of ^40^Ca + ^96^Zr and ^58^Ni + ^64^Ni are very similar to each other vs $E\sigma$, and are clearly lower than what observed for the other cases which are overlapping to a large extent. Once more, we note that inelastic couplings do not change the slope of the excitation functions, while strong transfer couplings do.

Looking in more detail, we note that the data of ^58^Ni + ^64^Ni are very near to those of the group of “inelastic” systems down to E$\sigma \approx$15 MeV mb, corresponding to E$/V_b\approx$0.93^21^. Below that, the points for this system have a fast decrease, leading to the overlap with ^40^Ca + ^96^Zr for lower $E\sigma$. It appears that transfer couplings determine the fusion dynamics only below that energy, for ^58^Ni + ^64^Ni. This is different from the case of ^40^Ca + ^96^Zr^20^ where the evidence is that such couplings dominate the full range of energies from the barrier down (see also Fig. 4).

**Table 1:** The width parameter $\hbar \omega$ obtained by fitting the excitation functions of several systems with the Wong formula.

System	$\hbar \omega$ (MeV)
^36^S + ^48^Ca	3.25
^48^Ca + ^48^Ca	3.23
^40^Ca + ^90^Zr	5.37
^40^Ca + ^96^Zr	10.1
^58^Ni + ^64^Ni	8.80
^64^Ni + ^64^Ni	3.13
^58^Ni + ^54^Fe	3.28
^16^O + ^208^Pb	3.07

We report in Table 1 the width parameters $\hbar \omega$ resulting from the fits of the measured excitation functions, using the Wong formula, for the systems shown in Fig. 10 and for ^36^S,^48^Ca + ^48^Ca. One sees the trend already observed in that figure, and that the width parameters of these two last cases are close to those of the other systems where inelastic modes are predominant. For the two systems where transfer couplings are important, we have $\epsilon$=$\hbar \omega /2\pi \approx$1.5 MeV, corresponding to a “thin” barrier, while for all other reported cases the barrier is “thick”.

## Summary and conclusions

Heavy-ion fusion reactions below the Coulomb barrier are an interesting tool for the study of quantum tunneling in the presence of intrinsic degrees of freedom. This work has been dedicated to the analysis of fusion excitation functions in that energy range, pointing out the details of those reactions which can be evidenced by comparative analyses of several systems, based on the combined observation of the energy-weighted excitation functions $E\sigma$ in relation to their first energy derivatives $d(E\sigma )/dE$. We have recalled the physical background of the Wong’s formula and its implications, in relation to the basic concepts of the coupled-channels model.

That derivative directly depends on the *s*-wave transmission coefficient and on the value of the barrier radius. We have pointed out that the representation of $d(E\sigma )/dE$ vs $E\sigma$, when comparing the behaviour of several systems, removes the basic differences due to the varying Coulomb barrier height, and is sensitive to the width of the barrier. The overview of many relevant heavy-ion systems clearly shows that, depending on the nuclear structure and the presence of strong transfer channels that representation can reveal characteristic features.

The first derivative $d(E\sigma )/dE$ does not essentially change when comparing systems where couplings to inelastic excitations are dominant, at variance with cases where strong transfer couplings with $Q>$0 are present, and produce shallower excitation functions. This agrees with basic predictions of the coupled-channel model, and is a useful way to complement the information one can obtain from the analysis of the barrier distributions. We remark that obtaining such distributions from the experimental data, on the other hand, implies extracting the second derivative of the excitation function, thus bringing to larger experimental uncertainties, especially above the barrier, in most cases. The behaviour of various C + Mg, Si systems indicates that the present analysis may be complicated by the existence of cross section oscillations.

We point out that, when measuring fusion excitation functions of systems where data are not yet available, the simple analysis presented in this work, which is only based of experimental data, is very useful to obtain information on the main features of sub-barrier fusion dynamics. As a consequence, it may properly address a following interpretation of the results within the CC model or other refined theoretical approaches.

## Figures and Tables

**Figure 11:**
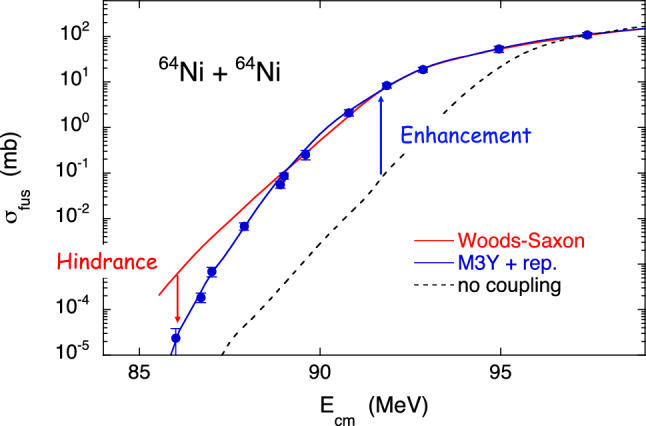
Fusion excitation function of ^64^Ni + ^64^Ni^23^ (see text).

**Figure 12:**
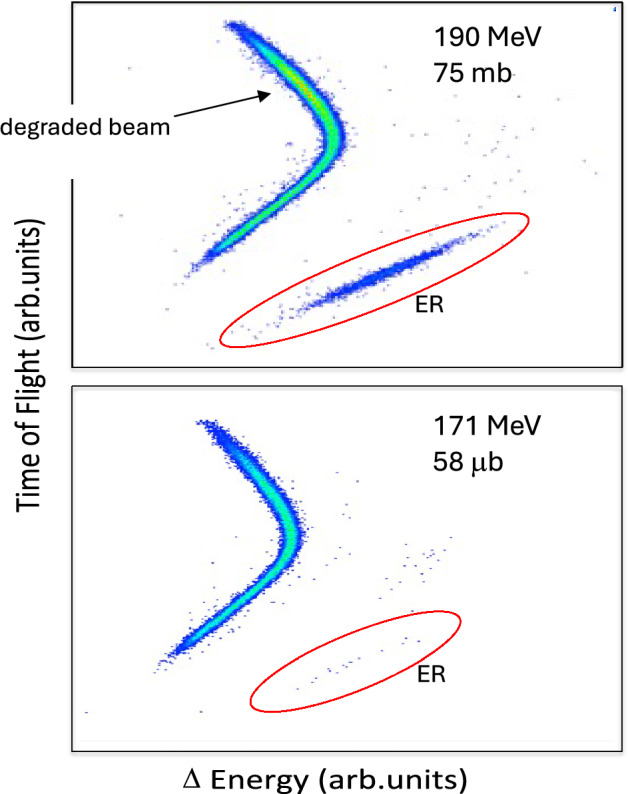
Two-dimensional plots *TOF* vs $\Delta E$ for ^58^Ni + ^64^Ni at two energies above and below the barrier^21^. The ER events are surrounded by the red curves and the cross sections are indicated.

## Data Availability

The experimental data presented here are available from the original articles listed in the References.

## Appendix

### The fusion hindrance effect

Starting from the first years of this millennium, it was found for many systems that, at deep sub-barrier energies, the cross section decreases very rapidly, so the excitation function is much steeper than the prediction of standard CC calculations. This phenomenon was called fusion hindrance. The first case where it was observed is ^60^Ni + ^89^Y^5^. Shortly after, hindrance was clearly identified is ^64^Ni + ^64^Ni^23^ as reported in Fig. 11. The cross sections were measured down to $\approx$ 25 nb and the hindrance threshold is around 0.1 mb. The asymmetric case of ^58^Ni + ^64^Ni^21^ does not show hindrance down to the measured level of a few $\mu$b.

The system ^40^Ca+^96^Zr has a behaviour similar to ^58^Ni + ^64^Ni. Its excitation function was measured down to $\simeq$2$\mu$b^20^ with a regular trend not evidencing any sign of hindrance. CC analyses performed for those two systems (see^21,41^ and Refs. therein) indicated that the absence of hindrance should be attributed to strong couplings to quasi-elastic neutron transfer with positive *Q*-values.

The origin of fusion hindrance is still much debated. Misicu and Esbensen^42,43^ adopted a double folding potential (“M3Y+repulsion”), producing a shallow pocket as a consequence of the incompressibility of nuclear matter. Alternatively, Ichikawa et al.^44^, proposed an adiabatic neck formation between the colliding nuclei in the overlap region. They used the Yukawa-plus-exponential potential^45^ and a damping of the coupling strengths inside the barrier is produced, leading to hindrance.

Recently, Simenel et al.^46^ have suggested that Pauli blocking is the mechanism underlying hindrance. They introduced a new microscopic approach to heavy ion fusion and showed that Pauli repulsion reduces the tunnelling probability inside the Coulomb barrier. They pointed out that, however, that in cases where the Q-values for nucleon transfer are large and positive, the valence nucleons can flow freely from one nucleus to the other without being hindered by the Pauli effect.

### The Wong’s formula

The Wong’s formula was derived^13^ by simple expressions for the total reaction cross section in terms of the interaction barrier for the *s* wave (Coulomb barrier), and it is a significant point of reference for research on heavy-ion fusion.

We recall the parabolic approximation of the ion-ion potential *V*(*r*) in the barrier $V_b$ region i.e.

4 $$\begin{aligned} V(r)=V_b-\frac{1}{2}\mu \omega ^2(r-R_b)^2 \end{aligned}$$

where $R_b$ is the barrier radius and $\hbar \omega$ is the curvature of the parabola

5 $$\begin{aligned} \hbar \omega =\sqrt{\frac{\hbar ^2}{\mu }\left| \frac{d^2V(r)}{dr^2}\right| _{R_b}} \end{aligned}$$

The quality of this approximation depends on the energy and the system mass. It is reasonable near the barrier and for heavy systems, becoming very inaccurate at energies far below the barrier. Hill and Wheeler^14^ obtained analytically the transmission coefficients through a parabolic barrier. The radius $R_l$ and the curvature $\hbar \omega _l$ of the barrier for the *l*-partial wave are only weakly dependent on *l*, so one can write

6 $$\begin{aligned} \hbar \omega _l\simeq \hbar \omega _o \hspace{5 mm} V_b (l)\simeq V_{b (o)}+\hbar ^2l(l+1)/2\mu R_{b(o)}^2 \end{aligned}$$

This led to the widely used Wong’s formula for the fusion cross section^13^

7 $$\begin{aligned} \sigma _{fus}^W (E)=\frac{\hbar \omega R_b^2}{2E}ln\left[ 1+exp\frac{2\pi }{\hbar \omega } (E-V_b)\right] \end{aligned}$$

which above the barrier, for $E>>V_b$, reduces to the classical formula

8 $$\begin{aligned} E\sigma _{fus}(E)=\pi R_b^2\left( E-V_b\right) \end{aligned}$$

Instead, below the barrier, the Wong’s formula is well approximated by the expression

9 $$\begin{aligned} E\sigma _{fus}(E)=\frac{\hbar \omega R_b^2}{2}exp\left[ \frac{2\pi }{\hbar \omega } (E-V_b)\right] \end{aligned}$$

because if $exp[\frac{2\pi }{\hbar \omega } (E-V_b)]<<1$, the cross section decreases exponentially with decreasing energy below the barrier.

### Measuring fusion cross sections

Several experimental set-ups and methods have been utilised to study the heavy-ion fusion reactions near and below the Coulomb barrier, presented before. For such measurements detectors with high efficiency, high-intensity and -quality beams with well-defined energies, and targets that can withstand those beams, are needed. A special effort has to be placed on understanding possible background effects, especially in the range of cross sections below $\approx$10 $\mu$b.

Most of the experimental results discussed in this article were obtained by detecting fusion-evaporation residues (ER). The difficulties associated with this method are related to the fact that ER are emitted at forward angles where the transmitted beam, together with beam-like particles and strong Rutherford scattering may prevent a clean identification and counting of the fusion events. Therefore the setup must be able to reject a large part of the beam particles. The ratio of incoming/transmitted beam particles is called the rejection factor of the setup. The rejection may be performed using electric and/or magnetic fields exploiting the different trajectories of ER and beam or beam-like particles. Fig. 12, as an example, shows the results of measurements of ^58^Ni + ^64^Ni fusion^21^, performed using the electrostatic deflector set-up of INFN-Laboratori Nazionali di Legnaro (LNL)^4^. The ER events are identified in two-dimensional plots of time-of-flight *TOF* vs energy loss $\Delta E$.

In measurements of sub-barrier fusion by ER detection, the target isotopic purity is essential. This is especially important when the target is the lightest stable nuclide of an isotopic chain. Even very small contamination of heavier isotopes will bring unwanted contributions to the fusion yields due mainly to the lower Coulomb barrier in the laboratory system, as pointed out in Ref.^3^.

Furthermore, the results of some experiments may be affected by ion beam impurities. This is not the case at accelerators using sputtering sources, but when Electron Cyclotron Resonance (ECR) ion sources are employed, one should take into account the possibility of having contaminations from previously used ion beams. Additional background can be produced by contaminations of a chemical nature in the target. Therefore, special care should be taken in the choice of the target material and in the procedure adopted to produce the targets. This is essential for experiments aiming at the measurement of very small cross sections.

## References

[1] Back BB, Esbensen H, Jiang CL, Rehm KE (2014). Recent developments in heavy-ion fusion reactions. Rev. Mod. Phys..

[2] Hagino K, Takigawa N (2012). Subbarrier fusion reactions and many-particle quantum tunneling. Prog. Theor. Phys..

[3] Jiang CL, Back BB, Rehm KE, Hagino K, Montagnoli G, Stefanini AM (2021). Heavy-ion fusion reactions at extreme sub-barrier energies. Eur. Phys. J. A.

[4] Montagnoli G, Stefanini AM (2023). Recent experimental results in sub- and near-barrier heavy ion fusion reactions (2nd edition). Eur. Phys. J. A.

[5] Jiang CL (2002). Unexpected behavior of heavy-ion fusion cross sections at extreme sub-barrier energies. Phys. Rev. Lett..

[6] Dasso CH, Landowne S, Winther A (1983). A study of Q-value effects on barrier penetration. Nucl. Phys. A.

[7] Dasso CH, Landowne S, Winther A (1983). Channel-coupling effects in heavy-ion fusion reactions. Nucl. Phys. A.

[8] Dasso CH, Landowne S, Winther A (1985). Barrier penetration in the presence of intrinsic degrees of freedom. Nucl. Phys. A.

[9] Dasgupta M, Hinde DJ, Rowley N, Stefanini AM (1998). Measuring barriers to fusion. Annu. Rev. Nucl. Part. Sci..

[10] Piasecki E (2009). Effects of weakly coupled channels on quasielastic barrier distributions. Phys. Rev. C.

[11] Piasecki E (2019). Dissipation and tunneling in heavy-ion reactions near the Coulomb barrier. Phys. Rev. C.

[12] Jiang CL, Rehm KE, Back BB, Esbensen H, Janssens RVF, Stefanini AM, Montagnoli G (2014). Influence of heavy-ion transfer on fusion reactions. Phys. Rev. C.

[13] Wong CY (1973). Interaction barrier in charged-particle nuclear reactions. Phys. Rev. Lett..

[14] Hill DL, Wheeler JA (1953). Nuclear Constitution and the Interpretation of Fission Phenomena. Phys. Rev..

[15] Dasso C.H. Effects of reaction channels in subbarrier fusion reactions, Proc. XXII Int. Winter Meeting on Nuclear Physics, Bormio, Italy, NORDITA–84/11(PREPR.) **21**, 22 (1984).

[16] Akyüz Ö. & Winther Å. in Nuclear Structure and Heavy-Ion Physics, Proceedings of the International School of Physics “Enrico Fermi”, Course LXXVII, Varenna, edited by R. A. Broglia and R. A. Ricci (North Holland, Amsterdam, 1981)

[17] Stefanini AM (2009). How does fusion hindrance show up in medium-light systems? The case of ^48^Ca + ^48^Ca. Phys. Lett. B.

[18] Stefanini AM (2008). Fusion of the positive Q-value system ^36^S + ^48^Ca well below the Coulomb barrier. Phys. Rev. C.

[19] Hagino K, Rowley N, Kruppa AT (1999). A program for coupled-channel calculations with all order couplings for heavy-ion fusion reactions. Comput. Phys. Comm..

[20] Stefanini AM (2014). Fusion of ^40^Ca + ^96^Zr revisited: Transfer couplings and hindrance far below the barrier. Phys. Lett. B.

[21] Stefanini AM (2019). Fusion hindrance and Pauli blocking in ^58^Ni + ^64^Ni. Phys. Rev. C.

[22] Timmers H (1998). A case study of collectivity, transfer and fusion enhancement. Nucl. Phys. A.

[23] Jiang CL (2004). Influence of nuclear structure on sub-barrier hindrance in Ni+Ni fusion. Phys. Rev. Lett..

[24] Timmers H (1997). Strong isotopic dependence of the fusion of ^40^Ca + ^96^Zr. Phys. Lett. B.

[25] Jia HM (2012). Fusion of the ^16^O + ^76^Ge and ^18^O + ^74^Ge systems and the role of positive Q-value neutron transfers. Phys. Rev. C.

[26] Liang, J. F. *et al.**Phys. Rev. Lett.* **91**, 152701 (2003). 10.1103/PhysRevLett.91.152701 14611462

[27] Liang, J. F. *et al.**Phys. Rev. C* **75**, 054607 (2007).

[28] Jiang, C. L. *et al.**Phys. Rev. C* **91**, 044602 (2015).

[29] Beckerman M, Salomaa M, Sperduto A, Molitoris JD, DiRienzo A (1982). Sub-barrier fusion of ^58,64^Ni with ^64^Ni and ^74^Ge. Phys. Rev. C.

[30] Morton CR (1999). Coupled-channels analysis of the ^16^O + ^208^Pb fusion barrier distribution. Phys. Rev. C.

[31] Dasgupta M (2007). Beyond the coherent coupled channels description of nuclear fusion. Phys. Rev. Lett..

[32] Stefanini AM (2010). Fusion hindrance for ^58^Ni + ^54^Fe. Phys. Rev. C.

[33] Stefanini AM (2017). New results in low-energy fusion of ^40^Ca + ^90,92^Zr. Phys. Rev. C.

[34] Stefanini AM (2013). Fusion of ^60^Ni + ^100^Mo near and below the Coulomb barrier. Eur. Phys. J. A.

[35] Jiang CL (2005). Hindrance of heavy-ion fusion at extreme sub-barrier energies in open-shell colliding systems. Phys. Rev. C.

[36] Stefanini AM (1992). Cross sections and mean angular momenta for ^64^Ni + ^92,96^Zr fusion near and below the Coulomb barrier. Nucl. Phys. A.

[37] Stefanini AM (2023). Sub-barrier fusion in ^12^C + ^26,24^Mg: Hindrance and oscillations. Phys. Rev. C.

[38] Montagnoli G (2020). Fusion of ^12^C + ^24^Mg far below the barrier: Evidence for the hindrance effect. Phys. Rev. C.

[39] Montagnoli G (2018). Fusion hindrance for the positive Q-value system 12C + 30Si. Phys. Rev. C.

[40] Esbensen H (2012). Structures in high-energy fusion data. Phys. Rev. C.

[41] Esbensen H, Montagnoli G, Stefanini AM (2016). Revised analysis of 40Ca+96Zr fusion reactions. Phys. Rev. C.

[42] Misicu S, Esbensen H (2007). Signature of shallow potentials in deep sub-barrier fusion reactions. Phys. Rev. C.

[43] Esbensen H, Misicu S (2007). Hindrance of 16O+208Pb fusion at extreme sub-barrier energies. Phys. Rev. C.

[44] Ichikawa T, Hagino K, Iwamoto A (2007). Existence of one-body barrier revealed in deep sub-barrier fusion. Phys. Rev. C.

[45] Krappe HJ, Nix JR, Sierk AJ (1979). Unified nuclear potential for heavy-ion elastic scattering, fusion, fission, and ground-state masses and deformations. Phys. Rev. C.

[46] Simenel C, Umar AS, Godbey K, Dasgupta M, Hinde DJ (2017). How the Pauli exclusion principle affects fusion of atomic nuclei. Phys. Rev. C.

